# Cataract surgery combined with micro-incision vitrectomy in patients with behcet’s disease uveitis

**DOI:** 10.1186/s12886-018-0813-3

**Published:** 2018-06-28

**Authors:** Fang Fan, Zhiyang Jia, Kejun Li, Xiaobin Zhao, Qingmin Ma

**Affiliations:** Department of Ophthalmology, Hebei general hospital, Shijiazhuang, Hebei 050000 People’s Republic of China

**Keywords:** Cataract surgery, Micro-incision vitrectomy, Behcet uveitis

## Abstract

**Background:**

This study sought to report the outcomes of a combined cataract extraction, intraocular lens (IOL) insertion and micro-incision vitrectomy (MIVS) procedure for the treatment of Behcet uveitis.

**Methods:**

This investigation involved the retrospective evaluation of a case series of patients with Behcet uveitis who underwent cataract extraction, IOL insertion and MIVS in a single surgical session at the same institution between January 2013 and November 2016. Outcome measures included visual acuity, inflammatory reaction, systemic anti-inflammatory medications, intraocular pressure (IOP) and complications.

**Results:**

Seven eyes of seven patients with a mean age of 39.00 ± 5.54 years (range, 32 to 48 years) and a mean follow-up duration of 13.57 ± 5.83 months (range, 6 to 24 months) were studied; five patients with a history of well-controlled uveitis were included. All patients underwent cataract extraction and IOL implantation combined with MIVS. All patients received postoperative steroids, which were slowly tapered during the weeks after surgery. There were no significant complications related to the surgery. Overall, best-corrected visual acuity (BCVA) was improved from log MAR (logarithm of the minimum angle of resolution) 1.67 ± 0.67 preoperatively to log MAR 0.74 ± 0.35 postoperatively; this improvement was statistically significant (*p* < 0.05). All eyes were deemed quiet at follow-up, and no patients required the escalation of therapy for long-term uveitis control.

**Conclusions:**

This retrospective series indicates that a procedure that combines phacoemulsification, IOL implantation and MIVS is a feasible technique for the removal of cataracts and pathologic vitreous in eyes with Behcet uveitis. This approach can restore vision without obvious complications.

## Background

Behcet uveitis is a common condition worldwide. This condition remains problematic because of its long course and therapeutic challenges. In combination with chronic persistent inflammation, it is also associated with a high rate of ocular complications and a high risk of permanent visual loss [1]. In certain cases, surgical treatment for Behcet uveitic eyes with cataract or posterior segment complications is inevitable. However, fear of surgery-associated intraocular inflammation has limited the clinical application of such treatment. Recently, there has been increasing progress in combined surgical techniques for cataract and pars plana vitrectomy surgeries, such as micro-incision cataract surgery combined with 23G or 25G sutureless vitrectomy (micro-incision vitrectomy (MIVS)) [2–4]. Besides, combined surgery has been complicated in eyes with uveitis [5, 6]. Thus, a procedure that combines phacoemulsification and MIVS may be a feasible treatment option in cases involving patients with Behcet uveitis accompanied by cataract and posterior segment complications.

In this study, we sought to evaluate the safety and tolerance of a procedure that combines phacoemulsification, IOL insertion and MIVS in a series of seven eyes with Behcet uveitis and to evaluate pre- and postoperative clinical factors associated with this condition.

## Methods

### Patients

We reviewed the medical records of patients suffering from Behcet uveitis who were treated in the Department of Ophthalmology of Hebei General Hospital between January 2013 and November 2016. This study adhered to the tenets set forth in the Declaration of Helsinki. Approval from the institution’s ethics committee was obtained prior to conducting this study.

All patients were diagnosed with Behcet disease based on the criteria issued by the International Study Group for Behcet’s Disease [7–9]. A subset of patients were receiving oral immunomodulatory therapy (cyclophosphamide and/or cyclosporine A). All patients underwent the collection of a detailed whole-body history, clinical evaluation and a comprehensive ocular examination that included the measurement of best-corrected visual acuities (BCVAs) (and conversion to the corresponding logarithm of the minimum angle of resolution (logMAR) values), slit-lamp examination, intraocular pressure (IOP) measurement, and fundus photography, if possible (FF 450plus; Carl Zeiss Meditec AG, Jena, Germany). Fluorescein angiography (FFA) and indocyanine green angiography (ICGA) were performed as required (FF 450plus; Carl Zeiss Meditec AG, Jena, Germany).Optical coherence tomography (OCT) (Cirrus HD-OCT 400, Carl Zeiss Meditec AG, Jena, Germany) and B-scan ultrasound evaluation (D0302; Meda Co., Ltd., TianJin, China) were performed during preoperative biometry, and a postoperative refraction of − 0.5 to 1.0 dioptres (D) was targeted. Whole-body examinations included determinations of typical clinical presentation, a tuberculin skin test, a chest X-ray or chest CT, a *Treponema pallidum* haemagglutination test, and serologic tests for HIV.

### Surgical technique and postoperative treatment

All patients were preoperatively notified about the risks of the surgery. The combined surgical procedures were performed by one experienced surgeon. All surgical interventions were conducted under local anaesthesia. Cataract surgery was performed first. After a scleral tunnel approach was used to create microincisions (2.8 mm) in the superior sclera, lysing posterior synechiae as needed, continuous curvilinear capsulorrhexis and cortical cleaving hydrodissection were performed. Phacoaspiration was established using a bi-manual technique. If possible, a one-piece foldable hydrophobic acrylic IOL (Rayner, Rayner, UK) was inserted into either the capsule or the ciliary sulcus. A 23 G microcannular vitrectomy system was used for the MIVS procedure. All microcannulas were inserted 3.5 mm posterior to the limbus. After the first microcannula was inserted in the inferotemporal quadrant for the infusion cannula, two additional microcannulas were inserted in the superonasal and superotemporal quadrants for the light pipe and vitrectomy probe. During the vitreous surgery, the vitreous and the epiretinal membranes were removed to the greatest possible extent, and the vitreous base was clearly ablated. In all subjects, preventive capsulotomy was performed using the vitrectomy probe. If necessary, eyes were tamponaded with silicon oil, C_3_F_8_ or sterile air at the conclusion of the surgical procedure. For all eyes, a subtenon injection of 3 mg dexamethasone was administered at the end of the operation. Topical steroid eye drops (prednisolone acetate 1%; Alcon, USA), topical eye drops containing the non-steroidal anti-inflammatory drug pranoprofen (Santen, Japan) and a systemic steroid (prednisolone 1 mg/kg/day) were administered to all patients after surgery. Systemic corticosteroid therapy was tapered based on patients’ conditions.

### Patient follow-up

All patients were evaluated at 1 day, 3 days, 7 days, and 1 month after surgery. Patients were evaluated every month thereafter. Preoperative and postoperative BCVAs were measured using a Snellen visual acuity chart; spherical equivalents, anatomic outcomes and complications were also evaluated. If necessary, FFA and OCT were performed.

### Statistical analysis

BCVAs were measured using a Snellen visual acuity chart and converted into logMAR values for statistical analysis. Mean values were compared using GraphPad Prism statistical software (La Jolla, CA). The threshold used for statistical significance was *p* < 0.05.

## Results

This study involved a total of 7 eyes from 6 male patients and 1 female patient. The mean patient age was 39.00 ± 5.54 years (range,32 to 48 years), and the mean follow-up duration was 13.57 ± 5.83 months (range, 6 to 24 months). The duration of Behcet disease before surgery was 6.50 ± 6.32 years (range, 0.5 to 20). Clinical characteristics of the patients are summarized in Table. 1. All patients experienced systematic symptoms of Behcet disease; these symptoms included oral ulcers (all patients), genital ulcers (4 patients, 57.14%), skin lesions (2 patients, 28.57%) and arthritis (2 patients, 28.57%). All patients suffered from recurrent attacks of anterior uveitis with exacerbation of this condition over time. All subjects had a history of posterior uveitis or panuveitis. All eyes exhibited preoperative cataract. Two patients with active inflammation were treated with intravenous methylprednisolone pulse therapy before surgery. One subject exhibited combined tractional and rhegmatogenous retinal detachment and underwent tamponade with silicon oil. In 6 eyes (85.71%), IOLs were implanted during surgery, with implantation in the capsule in 5 eyes (71.43%). In 1 eye with posterior capsular rupture during surgery, an IOL was inserted into the ciliary sulcus, with sutures used for intrascleral fixation. An IOL was not implanted into 1 eye because of severe retinal detachment. In the eye tamponaded with silicon oil, the silicon oil was removed 6 months after vitrectomy, and an IOL was implanted at that time. The retina remained attached after silicone oil removal.

**Table 1:** Patients’ characteristics, sugery, vision outcomes and complications

	Patient						
	1	2	3	4	5	6	7
Age	48	36	34	39	43	32	41
Gender	female	male	male	male	male	male	male
Eye	right	right	left	right	right	left	left
Duration of BD	20	0.5	6	5	3	4	7
Indications for surgery							
Cataract	+	+	+	+	+	+	+
PVO	+	+	+	+	+	+	+
RD	–	–	–	–	+	+	–
CME	+	–	+	–	–	–	+
Stage	active	stable	stable	stable	active	stable	stable
SystemTreatment							
Preoperative	CA + MPT	C A	–	–	CTX + MPT	–	CA
postoperative	OP	OP	OP	OP	OP	OP	OP
Surgical procedure	P + I + MIVS+C + ILMP+DA	P + I + MIVS+C + ILMP	P + I + MIVS+C + ILMP	P + I + MIVS+C + ILMP	P + I + MIVS+ILMP+C + EP + C3F8	P + MIVS+C + ILMP+ EP + SO	P + I + MIVS+C + ILMP
Follow up months	24	12	6	12	13	18	10
BCVA							
Preoperative	0.02	0.1	0.04	0.06	0.01	LP	0.04
Postoperative							
1 M	0.12	0.5	0.12	0.4	0.08	0.02	0.2
6 M	0.15	0.5	0.12	0.5	0.1	0.02	0.2
Last visit	0.12	0.6	0.12	0.5	0.1	0.08	0.2
CME							
Preoperative	+	–	+	–	–	–	+
Postoperative 1 M	+	+	+	+	+	+	+
Postoperative 6 M	–	–	–	–	–	–	–
IOL implant							
Capsule	+	+	–	+	+	–	+
Ciliary sulcus	–	–	+	–	–	+	–
Secondary surgery	–	–	–	–	–	+	–
Complications	posterior synechiae CE	CE	posterior capsular rupture CE	–	posterior synechiae CE	Mydriasis transient rise IOP CE	–

Retinal detachment: RD Persistent vitreous opacities: PVO cystoid macular edema: CME Cyclosporine A:CA methylprednisolone pulse therapy: MPT oral prednisolone:OP Cyclophosphamide:CTX micro-incision vitrectomy: MIVS capsulotomy: C endophotocoagulation: EP silicon oil:SO disinfect air:DA internal limiting membranepeeling: ILMP corneal edema: CE

The patients had a mean preoperative BCVA of logMAR 1.67 ± 0.67 (range, Light Perception (LP) (logMAR 3.0) to 20/200 (logMAR 1.0)) and a mean postoperative BCVA of logMAR 0.82 ± 0.48 (range, 20/1000 (logMAR 1.70) to 20/40 (logMAR 0.30)) at 6 months after surgery. A mean postoperative BCVA of logMAR 0.74 ± 0.35 (range, 20/250(logMAR 1.10) to 20/33 (logMAR 0.22)) at last visit. For the patient with retinal detachment, the final BCVA 6 months after silicon oil removal was 20/250. Cystoid macular oedema (CME) that was preoperatively confirmed using FFA and OCT was detected in 3 eyes (42.86%). However, postoperative CME was observed in all patients. For all patients, CME was relieved at 6-month follow-up evaluations. Owing to preventive capsulotomy performed using the vitrectomy probe, no patient exhibited posterior capsule opacification during follow-up. Complications during the operation included capsular rupture in one eye (14.29%); for this eye, an IOL was implanted in the ciliary sulcus, with sutures used for intrascleral fixation. The most common complication after surgery was transient corneal oedema, which was observed in 5 eyes (71.43%) but resolved without sequelae in all patients. Significant intraocular inflammation was observed in 3 eyes (42.86%), and posterior synechiae developed to different degrees in 2 eyes (28.57%); pupillary block did not occur. The patient who received silicon oil tamponade exhibited transient postoperative IOP elevation (range, 25 to 35 mmHg) that was effectively controlled using topical treatment. However, transient postoperative ocular hypotension was detected in this patient after silicon oil removal; this condition was alleviated within 2 weeks. During follow-up, all IOLs remained in situ, and IOL decentration did not occur. In this study, no patients had recurrent attacks of uveitis, and no subjects required the escalation of baseline therapy for the long-term control of intraocular inflammation.

## Discussion

Behcet disease, which presents as recurrent attacks of vasculitis involving small, medium and large blood vessels, is a multisystem disease [9]. Ocular involvement of Behcet disease with a dramatic impact on vision is typically bilateral and includes relapsing remitting panuveitis and a series of complications [10]. Reports have described the use of phacoemulsification with MIVS to treat complicated cataract and several posterior segment complications in patients with other types of chronic uveitis [6]. However, there exist only limited data on the use of combined phacoemulsification with MIVS for the management of cataract and coexisting posterior segment involvement in eyes affected by Behcet disease. The purpose of our research was to investigate an approach to treat anterior and posterior complications in eyes with Behcet uveitis.

After surgery, BCVA was improved in all eyes. Moderate uveitis control was maintained for most patients. Intraocular inflammation was observed with corneal oedema and anterior chamber inflammatory reaction; posterior synechiae developed to various extents in 2 eyes (28.57%). No eyes exhibited pupillary block. All patients developed CME 1 month after surgery; fortunately, CME had resolved by 6 months after surgery in all eyes. A possible mechanism underlying this phenomenon might be considerable intraoperative manipulation; the cataract surgery itself can disrupt the blood-aqueous barrier and produce susceptibility to CME in an eye with Behcet uveitis. However, the resolution of CME within 6 months after surgery might be attributable to the benefits of MIVS, internally limiting membrane peeling and systemic corticosteroid application. Cataract surgery is difficult in eyes with recurrent uveitis for iris depigmentation, lack of flexibility. The incidence of capsular rupture was high. In our study, posterior capsular rupture were observed in 1 eye during surgery.

To improve long-term uveitis control, satisfactory control of inflammation for at least 3 months prior to surgery is recommended for patients with uveitis. However, there exists research suggesting that cataract surgery and vitrectomy offer a surgical approach for clearing vitreous opacities and avoiding the release from the lens of inflammatory material with high permeation during a state of persistent inflammation [11]. This study included cases in which active inflammation was treated with intravenous methylprednisolone pulse therapy before surgery; however, inflammation after surgery was not severe. Consistent with this outcome, research has indicated that systemic interferon-alpha therapy resulted in less pronounced postoperative inflammation after intraocular surgery in subjects with ocular Behcet disease [12]. Surgery might be a method for relieving persistent inflammation. Combined surgery for eyes with chronic uveitis has produced encouraging results [5, 6, 13–15]. Our findings indicated the safety and utility of our combined procedure for Behcet uveitis. Traditionally, phacoemulsification and IOL implantation are always accomplished before or after vitrectomy. This typical approach generally requires two surgeons and two operations and therefore involves relatively high costs and long hospital stays. One-stage surgery could simultaneously resolve cataract and posterior segment involvement, thereby shortening patient discomfort, reducing costs and accelerating visual recovery. Our results obtained using combined phacoemulsification, IOL implantation and MIVS in a single surgery confirm the efficiency of this technique, which has been described in previous reports [13–16]. Moreover, MIVS improves operative efficiency and is a relatively non-invasive surgery with an acceptable complication rate and mild postoperative inflammatory reactions. This procedure may be a reasonable option for eyes with Behcet uveitis that concurrently exhibit cataract and posterior changes. Such eyes are prone to serious postoperative inflammation if treated with large instruments and more surgical manipulations. A technique involving scleral tunnel phacoemulsification and IOL implantation followed by MIVS was used in our study. In our experience, this procedure has been extremely promising. A scleral tunnel can avoid corneal wound leakage during vitrectomy; furthermore, better corneal transparency can reduce the difficulty of vitrectomy. In our opinion, the technique of IOL implantation followed by MIVS is relatively easy and requires a short operation time. We used viscoelastic material when the posterior surface of an IOL was watered during gas–fluid exchange. However, whether IOL implantation was performed was dependent on the patient’s condition. There were no complications during surgery. We implanted an IOL into the capsular bag to the greatest possible extent. In our study, visual acuity was improved in all patients after surgery. The longest follow-up time was 2 years. Only two patients developed obvious iris adhesion, and no secondary glaucoma or recurrence of uveitis was observed. Our study had several limitations, including an inadequate number of cases and limited follow-up time. Further investigation is needed.

## Conclusions

This retrospective series indicates that a combined phacoemulsification, IOL implantation and MIVS procedure appears to improve BCVA and can be an effective and safe intervention for treating patients with Behcet uveitis with cataract and vitreoretinal complications. This technique also has beneficial effects on the long-term course of Behcet uveitis and may restore or preserve vision. However, more case series with longer follow-up periods are needed to identify the natural course of Behcet uveitis and to evaluate the outcomes of this technique.

## Abbreviations

BCVA: Best-corrected visual acuity
CME: Cystoid macular edema
FFA: Fluorescein angiography
ICGA: Indocyanine green angiography
IOL: Intraocular lens
IOP: Intraocular pressure
MIVS: Micro-incision vitrectomy
OCT: Optical coherence tomography
SE: Spherical equivalents
TST: Tuberculin skin test
